# T-to-R switch of muscle fructose-1,6-bisphosphatase involves fundamental changes of secondary and quaternary structure

**DOI:** 10.1107/S2059798316001765

**Published:** 2016-03-30

**Authors:** Jakub Barciszewski, Janusz Wisniewski, Robert Kolodziejczyk, Mariusz Jaskolski, Dariusz Rakus, Andrzej Dzugaj

**Affiliations:** a Center for Biocrystallographic Research, Institute of Bioorganic Chemistry, Polish Academy of Sciences, Poznan, Poland; b Department of Animal Molecular Physiology, Wrocław University, Wrocław, Poland; c Department of Crystallography, Faculty of Chemistry, A. Mickiewicz University, Poznan, Poland; d Department of Genetics, Wrocław University, Wrocław, Poland

**Keywords:** allostery, AMP binding, energy metabolism, fructose-1,6-bisphosphatase, gluconeogenesis, quaternary transformations

## Abstract

Fructose-1,6-bisphosphatase (FBPase) catalyzes the hydrolysis of fructose 1,6-bisphosphate to fructose 6-phosphate and is a key enzyme of gluconeogenesis and glyconeogenesis and, more generally, of the control of energy metabolism and glucose homeostasis. Vertebrates, and notably *Homo sapiens*, express two FBPase isoforms. The liver isozyme is expressed mainly in gluconeogenic organs, where it functions as a regulator of glucose synthesis. The muscle isoform is expressed in all cells, and recent studies have demonstrated that its role goes far beyond the enzymatic function, as it can interact with various nuclear and mitochondrial proteins. Even in its enzymatic function, the muscle enzyme is different from the liver isoform, as it is 100-fold more susceptible to allosteric inhibition by AMP and this effect can be abrogated by complex formation with aldolase. All FBPases are homotetramers composed of two intimate dimers: the upper dimer and the lower dimer. They oscillate between two conformational states: the inactive T form when in complex with AMP, and the active R form. Parenthetically, it is noted that bacterial FBPases behave somewhat differently, and in the absence of allosteric activators exist in a tetramer–dimer equilibrium even at relatively high concentrations. [Hines *et al.* (2007 ▸), *J. Biol. Chem.* **282**, 11696–11704]. The T-to-R transition is correlated with the conformation of the key loop L2, which in the T form becomes ‘disengaged’ and unable to participate in the catalytic mechanism. The T states of both isoforms are very similar, with a small twist of the upper dimer relative to the lower dimer. It is shown that at variance with the well studied R form of the liver enzyme, which is flat, the R form of the muscle enzyme is diametrically different, with a perpendicular orientation of the upper and lower dimers. The crystal structure of the muscle-isozyme R form shows that in this arrangement of the tetramer completely new protein surfaces are exposed that are most likely targets for the interactions with various cellular and enzymatic partners. The cruciform R structure is stabilized by a novel ‘leucine lock’, which prevents the key residue, Asp187, from locking loop L2 in the disengaged conformation. In addition, the crystal structures of muscle FBPase in the T conformation with and without AMP strongly suggest that the T-to-R transition is a discrete jump rather than a shift of an equilibrium smooth transition through multiple intermediate states. Finally, using snapshots from three crystal structures of human muscle FBPase, it is conclusively demonstrated that the AMP-binding event is correlated with a β→α transition at the N-terminus of the protein and with the formation of a new helical structure.

## Abstract 2

When crystallized in the absence of the allosteric inhibitor AMP, human muscle fructose-1,6-bisphosphatase has a totally unexpected quaternary structure of its active R form, with the two dimers of the homotetrameric molecule in a perpendicular orientation, in stark contrast to the coplanar arrangement of the closely related liver isozyme. The T-to-R switch of the muscle enzyme also involves a highly unusual α→β refolding of the N-terminus.

## 1. Introduction   

Fructose-1,6-bisphosphatase (FBPase; EC 3.1.3.11), which catalyzes the hydrolysis of fructose 1,6-bisphosphate to fructose 6-phosphate and inorganic phosphate, is a key enzyme of gluconeogenesis and glyconeogenesis (Tejwani, 1983 ▸) and, more generally, of the control of energy metabolism and glucose homeostasis. Vertebrate genomes contain two distinct genes, *FBP1* and *FBP2*, coding for two FBPase isozymes. Liver FBPase, the protein product of the *FBP1* gene, is expressed mainly in gluconeogenic organs, where it functions as a regulator of glucose synthesis from noncarbohydrates (Al-Robaiy & Eschrich, 1999 ▸). The activity of liver FBPase is regulated by fructose 2,6-bisphosphate, a compound whose concentration is under hormonal control (Bartrons *et al.*, 1983 ▸; Pilkis *et al.*, 1995 ▸) and which inhibits the liver enzyme synergistically with AMP (Van Schaftingen & Hers, 1981 ▸; Benkovic & deMaine, 1982 ▸; Liu & Fromm, 1988 ▸). Unlike liver FBPase, the muscle isoform, encoded by *FBP2*, is expressed in all vertebrate cells, both in glyconeogenic cells (*e.g.* muscle fibres) as well as in cells such as neurons which are not supposed to synthesize glycogen from carbohydrate precursors (Löffler *et al.*, 2001 ▸). Muscle FBPase is also present in cells that predominantly express the liver isozyme, *e.g.* in the liver itself (Al-Robaiy & Eschrich, 1999 ▸). Recent studies have demonstrated that the physiological role of muscle FBPase goes beyond its enzymatic function, as this isozyme was localized inside cell nuclei (Gizak *et al.*, 2009 ▸) and was shown to interact with mitochondria (Gizak *et al.*, 2012 ▸), where it is involved in regulation of the cell cycle (Gizak *et al.*, 2006 ▸; Mamczur *et al.*, 2012 ▸) and apoptosis (Gizak *et al.*, 2012 ▸; Pirog *et al.*, 2014 ▸), respectively. This moonlighting function of muscle FBPase does not rely on the catalytic activity of the enzyme but on its ability to interact with various nuclear and mitochondrial proteins, such as helicases, Myb-binding protein 1A, ribonucleoproteins (Mamczur *et al.*, 2012 ▸), ATP synthase sub­units, VDACs (voltage-dependent anion channels) and Slc25a5 (solute carrier family 25; adenine nucleotide translocator) (Gizak *et al.*, 2012 ▸).

Despite the high level of similarity of their primary structures, the two FBPase isozymes play entirely different biological functions and differ significantly in kinetic properties such as susceptibility to inhibition by AMP, NAD^+^ and Ca^2+^ (Dzugaj, 2006 ▸). Mammalian muscle FBPase is about 100 times more susceptible to the allosteric inhibitors AMP and NAD^+^ (Rakus *et al.*, 2003 ▸) and about 1000 times more sensitive to inhibition by Ca^2+^ than the liver isozyme (Gizak *et al.*, 2004 ▸, 2008 ▸; Zarzycki *et al.*, 2007 ▸), and it has been hypothesized that it may be catalytically active only upon association with the muscle isoform of aldolase (Rakus *et al.*, 2004 ▸). In line with this, calcium cations not only strongly inhibit muscle FBPase but also disrupt the Z-line-based muscle FBPase–muscle aldolase complex in striated muscles, blocking the re-synthesis of glycogen during high-exertion exercise (Gizak *et al.*, 2013 ▸; Rakus *et al.*, 2013 ▸). While the mechanism of the different sensitivity of muscle and liver FBPases to Ca^2+^ has been shown to depend on a single E69Q point mutation (Zarzycki *et al.*, 2007 ▸; Rakus *et al.*, 2013 ▸), the structural differences leading to the unique responses to allosteric inhibitors and differing abilities to interact with various binding partners are poorly understood.

**Figure 1:**
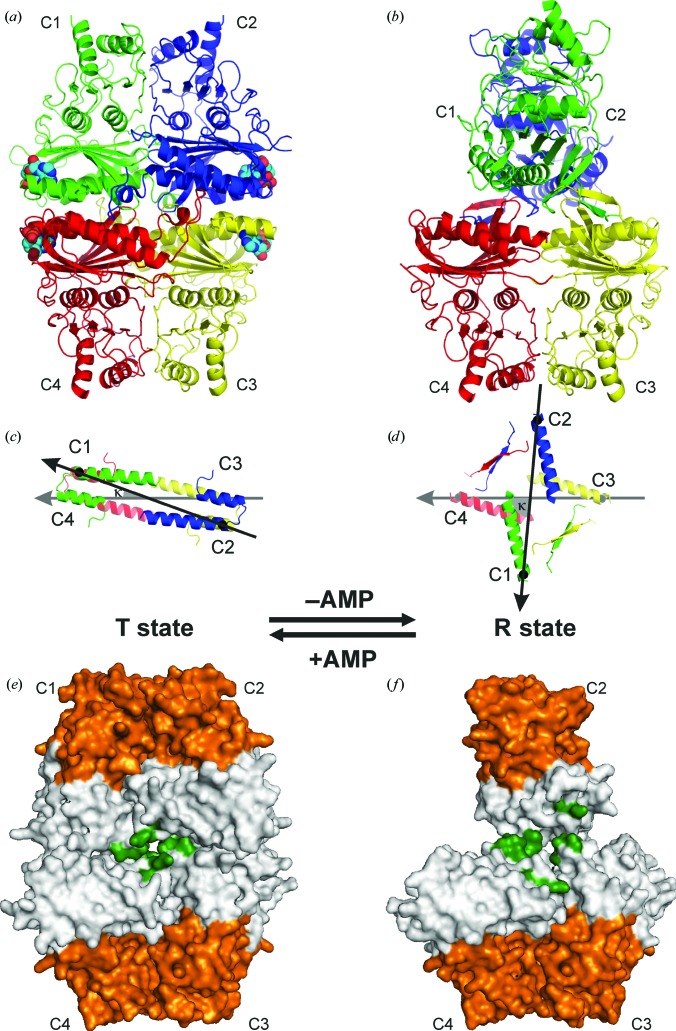
Overall fold of the muscle FBPase tetramer in the T (*a*) and R (*b*) states shown in cartoon representation with subunits, labelled C1–C4, in different colours. The secondary-structure elements are assigned according to *DSSP* (Kabsch & Sander, 1983 ▸). Four AMP molecules, shown as space-filling models, are present, one in each nucleotide-binding site of the T form. (*c*) and (*d*) show a top view of the structures in (*a*) and (*b*), respectively, with the definition of the κ angle (see text) between the vectors joining the C^α^ atoms of Leu30 (black or grey dots) in subunits C2→C1 (black arrow) and C3→C4 (grey arrow). (*e*, *f*) Overall architecture of the muscle FBPase tetramer in the T (*e*) and R (*f*) states, shown in surface representation. Each subunit is divided into two domains: the allosteric domain (grey) and the catalytic domain (orange). Hydrophobic residues forming the central cavity in the middle of the tetramer are marked in green. To show the details of the central hydrophobic cavity, the catalytic loop L2 has been omitted in the T state (*c*), while in the R state (*d*) the forefront subunit C1 is not shown.

Mammalian FBPase is a 222-symmetric homotetramer (Ke *et al.*, 1991 ▸) that is better described as a dimer of two dimers, referred to as the ‘upper’ (subunits C1 and C2) and ‘lower’ (subunits C3 and C4) dimers in Fig. 1 ▸(*a*). The 222 (*D*_2_) symmetry consists of three mutually perpendicular twofold axes. In the standard viewing direction, with the four subunits labelled C1–C2–C3–C4 in a clockwise manner starting with C1 at the upper-left corner, the dyad along *z* is oriented vertically (running up the page) and the dyads along *x* and *y* are in the equatorial plane, separating the upper dimer C1·C2 from the lower dimer C3·C4. The lower dimer C3·C4 is always seen with subunit C4 (left) viewed on strand β1 of the central β-sheet, and subunit C3 (right) viewed on β7. Typical quaternary transitions of the tetramer involve rotations (described by the angle κ) of the upper dimer relative to the lower dimer, but the mutual relation of the subunits within each dimer (*i.e.* within C1·C2 and C3·C4) is constant. In our convention, κ is defined as the angle between the vectors connecting the C^α^ atoms of Leu30 in subunits C3→C4 (the first vector) and C2→C1 (the second vector) after their projection onto the equatorial plane perpendicular to the *z* axis (Figs. 1 ▸ *c* and 1 ▸ *d*). A rotation of C2→C1 with respect to a stationary C3→C4 dimer, when viewed from the positive direction of the *z* axis (*i.e.* from C1·C2 towards C3·C4), is positive for clockwise rotations and negative for counterclockwise rotations. During such rotations, the individual dimers (C1·C2 and C3·C4) exactly preserve their *z*-axis symmetry as they rotate around this axis itself. Any *z*-axis rotation of this type will of course preserve the *D*_2_ symmetry, but will change the orientation (in the equatorial plane) of the *x* and *y* axes, which follow the subunit rotation with half of its amplitude. In the reference orientation with angle κ = 0° and the stationary C3→C4 vector aligned with the –*x* direction, the upper dimer C1·C2 has a face-on view, with subunit C1 on the left facing the viewer with strand β7, and subunit C2 on the right facing the viewer with β1. Since the *D*_2_-symmetric tetramer is a chiral object, subunits C2 and C4 are always viewed from their β1 side (‘front’) and the C1 and C3 subunits are viewed from the opposite direction (‘back’).

Our knowledge about the mechanism of FBPase is nearly fully based on studies of the enzyme isolated from porcine liver, while information about the muscle isoform is scarce and is limited to only five structural studies (Zarzycki *et al.*, 2011 ▸; Shi *et al.*, 2013 ▸). According to literature data, FBPase exists in at least two quaternary states, designated as R (catalytically active) and T (inactive), depending on the concentrations of the enzyme effectors (Ke *et al.*, 1991 ▸; Zhang *et al.*, 1994 ▸). Recombinant liver FBPase has a planar R state with κ ≃ 0°. In this idealized situation the four flat (slab-like) subunits lie in the *yz* plane; the *x* axis in the standard orientation points toward the viewer and the *y* axis runs across from left to right to preserve a right-handed axial system. In the liver FBPase T state the upper dimer is rotated by κ ≃ 13° relative to the lower dimer within the tetramer (Choe *et al.*, 2000 ▸).

A proposed mechanism governing the regulation and catalysis of liver FBPase involves three conformational states of a catalytic loop (labelled loop L2 in the topology inventory of the protein), called the engaged, disengaged and disordered states (Choe *et al.*, 1998 ▸). The enzyme is catalytically active if the loop can switch between the engaged and disordered conformations (Choe *et al.*, 1998 ▸, 2000 ▸; Nelson *et al.*, 2000 ▸). It was hypothesized that the catalytic loop can adopt the engaged conformation only in the R state of the tetramer (Choe *et al.*, 1998 ▸, 2000 ▸). Binding of AMP to liver FBPase induces the conversion of the enzyme to the T state, which is presumed to stabilize the disengaged, inactive conformation of the catalytic loop (Choe *et al.*, 1998 ▸, 2000 ▸; Nelson *et al.*, 2000 ▸).

Our previous study revealed that the structure of inactive (T-state), AMP-saturated muscle FBPase is practically identical to that of the T-state liver isozyme (Zarzycki *et al.*, 2011 ▸) and hence we suggested that the explanation of the markedly different properties of the two isoforms must lie in differences between the quaternary arrangements of the active R states of the enzymes. We hypothesized that the R state of muscle FBPase must vary considerably from that of the liver isozyme, for example, to present new binding surfaces for interaction with allosteric partners and to enable the R-to-T transition at a lower AMP concentration (Rakus *et al.*, 2004 ▸).

In the present study, we report the first crystal structure of muscle FBPase in its active R state. The structure reveals a picture that is diametrically different from the situation known from liver FBPase, with the upper and lower dimers forming a cruciform structure characterized by κ = −85° (Figs. 1 ▸ *b* and 1 ▸ *d*). The sense of the rotation could be deduced from steric hindrance factors involving the catalytic loop. In this completely novel R configuration of muscle FBPase, the C1 subunit has rotated towards the viewer relative to the stationary C3·C4 lower dimer. The differences in the quaternary structures of the R states between the two isozymes suggest new interaction surfaces for the recruitment of muscle FBPase-specific binding partners. In addition, by determining the structures of the wild-type muscle enzyme in the T state in complex with AMP [T(+AMP)] and with the cofactor removed by specific backsoaking [T(−AMP)], we also demonstrate that the transition from the inactive, AMP-associated T state towards the active R state involves a reversible refolding of a key helix that is part of the allosteric centre of muscle FBPase. Such a profound structural transformation has not been described for conservative closely related metabolic enzymes before and is therefore a novelty in structural enzymology.

## 2. Methods   

### 2.1. Protein crystallization   

Expression and purification of human muscle FBPase was carried out as described previously (Rakus *et al.*, 2003 ▸). AMP was removed from protein samples by extensive dialysis against 25 m*M* HEPES buffer (pH 7.0, 4°C), followed by FPLC gel filtration on a HiLoad Superdex 200 16/60 column (GE Healthcare) equilibrated with 100 m*M* MgCl_2_. The fractions containing FBPase were dialyzed to reduce the magnesium concentration to 5 m*M*.

Crystallization experiments were carried out at 292 K using the hanging-drop vapour-diffusion method. 3 µl drops were made by mixing a 1:1 volume ratio of the protein and precipitant solutions. Catalytically active wild-type FBPase without any ligands was concentrated to 6 mg ml^−1^ and crystallized using 10 m*M* Tris buffer pH 7.4 containing 10 m*M* MgCl_2_, 2 *M* NaCl and 10%(*v*/*v*) PEG 6000. Wild-type FBPase in complex with AMP at a concentration of 8 mg ml^−1^ in 25 m*M* HEPES buffer with 2 m*M* MgCl_2_ and 0.6 m*M* AMP was crystallized at pH 7.0 using 1.8 *M* ammonium citrate as the precipitating agent. The best crystals of FBPase with and without AMP (0.20 × 0.15 × 0.15 mm) grew within three months.

### 2.2. T-to-R transition   

For backsoaking of AMP, the crystals of FBPase in complex with AMP were transferred for 10 s to mother liquor supplemented with 100 m*M* MgCl_2_ and 20%(*v*/*v*) glycerol and were subsequently flash-vitrified at 100 K in a cold nitrogen gas stream.

### 2.3. X-ray data collection and processing   

Before the diffraction experiments, the crystals were cryoprotected in mother liquor supplemented with 20–30%(*v*/*v*) glycerol and then flash-vitrified at 100 K in a cold nitrogen-gas stream. X-ray diffraction data were collected for three crystals: (i) without AMP (1.67 Å resolution), (ii) with AMP (1.84 Å) and (iii) with AMP backsoaked using an MgCl_2_ solution (2.99 Å) (see §[Sec sec2.2]2.2). In all cases, synchrotron radiation was utilized as provided by the beamlines of the BESSY II synchrotron, Berlin, Germany equipped with Rayonix MX-225 square CCD detectors. The diffraction data were processed and scaled with *XDSAPP* (Kabsch, 2010 ▸; Krug *et al.*, 2012 ▸). Data-collection statistics are presented in Table 1 ▸.

### 2.4. Structure determination and refinement   

Muscle FBPase crystallized in two different space groups as follows: the active protein in the R state in space group *I*4_1_22 and the inactive protein in the T state in space group *C*222. The crystal structure of the T state is isomorphous to that described previously (Zarzycki *et al.*, 2011 ▸) for the E69Q mutant of human muscle FBPase (PDB entry 3ifa). It consists of two independent FBPase dimers (C1–C3 type), from which crystallographic symmetry generates two full tetramers. The crystal structure of the R form was solved by molecular replacement (MR) using *Phaser* (McCoy *et al.*, 2007 ▸) with chain *A* of the T form as the model. The asymmetric unit of this form contains only one protein molecule, from which the 222 site symmetry generates the complete tetramer. In several rounds of manual rebuilding in *Coot* (Emsley *et al.*, 2010 ▸) the models were corrected according to the electron-density maps, with special emphasis on the N-terminal fragment of the protein molecules. Refinement of all structures was carried out in *phenix.refine* (Afonine *et al.*, 2012 ▸). Four AMP molecules were modelled in the T-state structure designated T(+AMP). Riding H atoms of the protein molecules were included in *F*_c_ calculations for the R state and T(+AMP) structures. For all three models, *i.e.* the R state and the T state with [T(+AMP)] and without [T(−AMP)] AMP, TLS parameters (Winn *et al.*, 2001 ▸) were refined for six, four and four groups per subunit, respectively, as suggested by the refinement program. Water molecules were added to the R-state and T(+AMP) structures. The refinement statistics are presented in Table 1 ▸.

### 2.5. Database depositions   

The structures described in this paper, together with the structure-factor data, have been deposited in the Protein Data Bank under accession codes 5et6 (T-state muscle FBPase with AMP), 5et7 (T-state muscle FBPase without AMP) and 5et5 (R-state muscle FBPase). The corresponding raw X-ray diffraction images have been deposited in the RepOD Repository at the Interdisciplinary Centre for Mathematical and Computational Modelling (ICM) of the University of Warsaw, Poland and are available for download with the following digital object identifiers (DOIs): http://dx.doi.org/10.18150/8324764 [T(+AMP)], http://dx.doi.org/10.18150/10.18150/2374334 [T(−AMP)] and http://dx.doi.org/10.18150/6428373 (R).

### 2.6. CD spectra   

UV CD spectra of human muscle and liver FBPases were recorded on a Jasco J-815 spectrometer in the wavelength range 195–265 nm. The measurements were recorded using a 2 mm light-path-length cell with a step resolution of 1 nm at temperatures of 25, 35, 45, 55, 60, 65, 70 and 80°C. For each temperature the sample was equilibrated for 5 min and the spectrum was obtained by averaging three scans and subtracting the buffer signal. Before measurement, the protein samples were dialyzed against 25 m*M* phosphate buffer pH 7.4 at 25°C. The final protein concentration was 100 µg ml^−1^ for muscle FBPase and 50 µg ml^−1^ for the liver isozyme. Various cofactor-dependent conformational states of FBPase were obtained by the addition of Mg^2+^ ions at 5 m*M* for the R state or of AMP at 300 µ*M* for the T state. The raw data were interpreted and expressed as mean residue molar ellipticity in deg cm^2^ dmol^−1^.

### 2.7. Turbidimetric determination of the FBPase melting temperature   

Protein precipitation was assessed by measurement of light scattering at 600 nm wavelength using an Agilent 8453 UV–Vis spectrophotometer. The light path length was 10 mm. The measurements were conducted at temperatures ranging from 35 to 80°C in 2°C steps. Samples were equilibrated for 2 min before each measurement. To avoid sedimentation, the samples were stirred at 200 rev min^−1^. Buffer, protein and additive concentrations were the same as in the CD experiments (§[Sec sec2.6]2.6). An additional control experiment with 5 m*M* MgCl_2_ and without protein was performed to assess magnesium phosphate precipitation and to correct data from experiments containing MgCl_2_. Curves were fitted to experimental data points using the Agilent *UV-Vis ChemStation* software. To find the melting temperatures, derivative curves of absorbance *versus* temperature were calculated. Melting temperatures were calculated with the Agilent *UV-Vis ChemStation* software using the following method. Firstly, a set of equally spaced absorbance *versus* temperature data points with a temperature step of 0.1°C was created from the experimental data using linear interpolation. Next, derivative curves were calculated using the Savitzky–Golay filter (Savitzky & Golay, 1964 ▸) with a filter length of 21 and a polynomial degree of 2. The maxima of the derivative curves correspond to the melting temperatures.

## 3. Results   

### 3.1. The muscle FBPase tetramers   

Inactive FBPase in the T state in complex with AMP [T(+AMP)] crystallized in space group *C*222 isomorphously with the structure reported previously for the E69Q mutant of human muscle FBPase by Zarzycki *et al.* (2011 ▸). The asymmetric unit contains two independent dimers, chains A–B and C–D, corresponding to the C1–C3 subunits of the tetrameric enzyme. Crystallographic twofold axes along *x* (in the case of A–B) and *y* (C–D) generate two full tetramers (A·A′–B·B′ and C·C′–D·D′, respectively). Both tetramers therefore have partial crystallographic as well as partial noncrystallographic (NCS) symmetry. In both cases, the exact crystallographic symmetry generates the tight C1·C2-type dimers, which have an invariant structure that is not affected by the quaternary transformations of the tetramers (rotations of the entire C1·C2 units). The two tetramers in the *C*222 structure of the AMP complex are very similar. For example, superposition of the C–D dimer on the A–B dimer is characterized by an r.m.s.d. of 0.201 Å for 4352 superposed C^α^ atoms. The κ angles are identical: +20° for the A·A′–B·B′ and C·C′–D·D′ tetramers (Fig. 1 ▸ *a*). In each subunit, the AMP ligand had very good definition in *F*_o_ − *F*_c_ electron-density maps phased by the protein atoms only, and could be modelled without any ambiguity. In view of the above similarity, in the following analysis the A·A′–B·B′ tetramer will be used to represent the wild-type FBPase in the T state, unless stated otherwise. Some amino-acid residues in the T state with AMP (residues 1–9, 64–70, 142–145 and 335–337) and the T state without AMP (residues 1–10, 18–28, 51–71, 105–109, 123–129 and 335–337) were not modelled because of poor electron density.

The active R-state muscle FBPase forms crystals with *I*4_1_22 symmetry with one protein molecule (A or C1) in the asymmetric unit, from which the crystallographic symmetry generates the full tetramer with a κ angle of −85° (Fig. 1 ▸ *b*). The entire majority of the FBPase polypeptide chain could be modelled with full confidence, except for some amino-acid residues (1–7, 20–28, 52–69, 123–130, 141–147 and 332–337) which were disordered and thus were omitted from the model.

**Figure 2:**
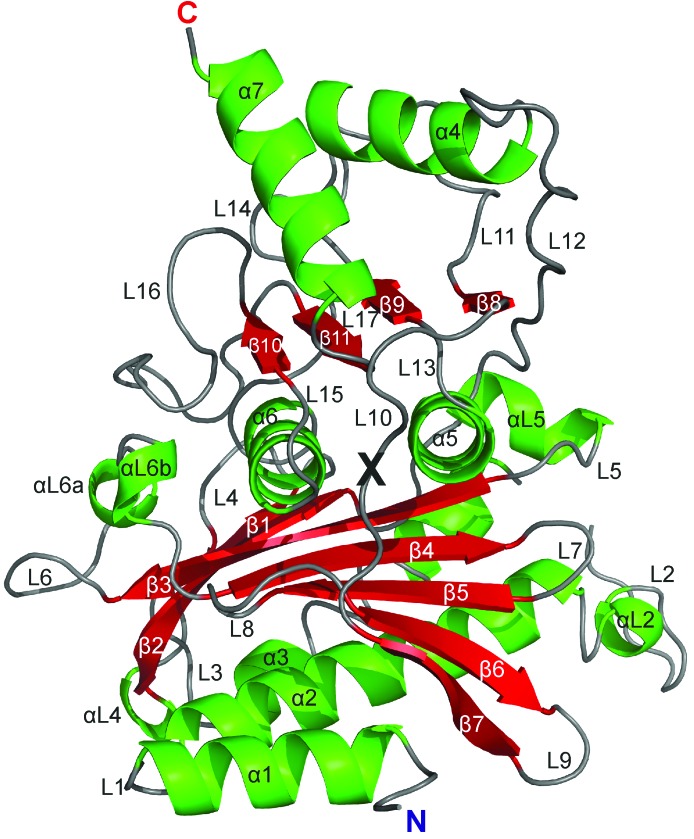
Overall fold of muscle FBPase subunit C1 in the T state, shown in cartoon representation with secondary-structure elements, as assigned according to *DSSP* (Kabsch & Sander, 1983 ▸), marked with different colours and labelled. The border residue Val200 between the allosteric and catalytic domains is marked with a black X.

Both the R-state and the T-state FBPases are homotetramers composed of upper (C1·C2) and lower (C3·C4) intimate dimers. Each protomer consists of two segments: the allosteric domain created by residues 1–200 and the catalytic domain created by residues 201–337 (Table 2 ▸). The border between the domains is located in the middle of loop L10 (Fig. 2 ▸). In the tetramer, the intimate dimers interact through surfaces of the allosteric domains. At the junction of the four sub­units in the middle of the tetramer there is a hydrophobic cavity surrounded by residues from the C-terminus of helix α2, the N-terminus of loop L2 and loop L8 from each subunit (Figs. 1 ▸ *e* and 1 ▸ *f*). This area is very conserved and is present in all known FBPases. Structural studies of the liver isozyme showed that the central cavity plays an important role in the T-to-R transition (Gao *et al.*, 2014 ▸).

### 3.2. The AMP-binding site   

**Figure 3:**
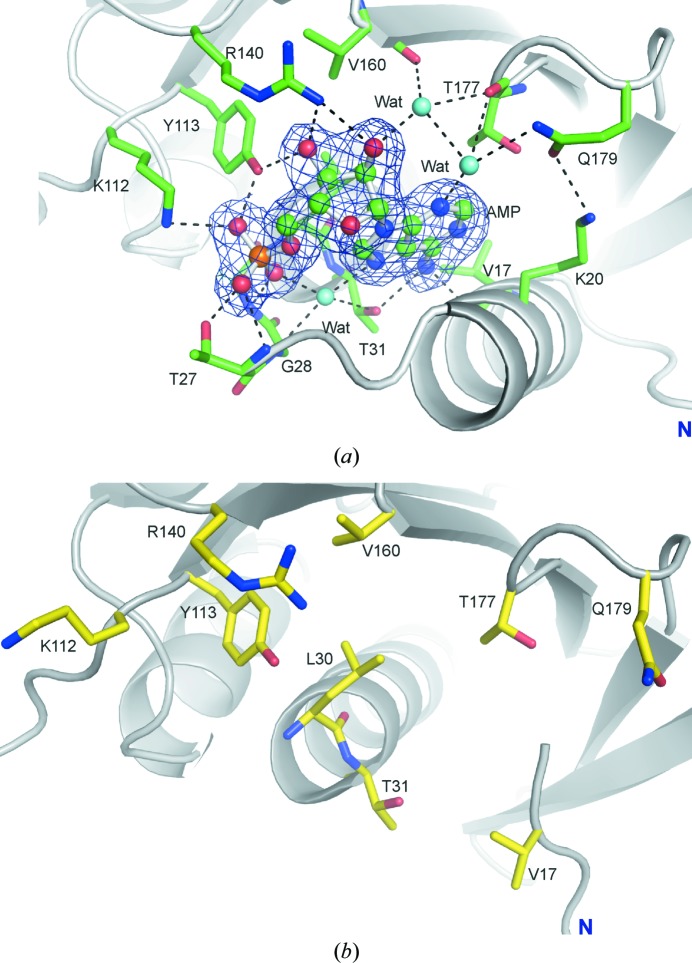
(*a*) The AMP-binding site of human muscle FBPase in the T state. The AMP molecule is shown in ball-and-stick representation. The *F*_o_ − *F*_c_ OMIT electron-density map (blue mesh) is contoured at the 4σ level. Hydrogen bonds are shown as dashed lines. (*b*) The AMP-binding site area in the R state, where no AMP is bound.

Each FBPase subunit in the T(+AMP) state binds one molecule of AMP with full occupancy. The base moiety of the nucleotide interacts with Val17 and Thr31 (Fig. 3 ▸ *a*). The ribose is recognized by the side chains of Tyr113 and Arg140 and by the main chain of Val160 and Thr177 *via* a water molecule. The O atoms of the phosphate group interact with the side chains of Thr27, Lys112 and Tyr113 and with the main chains of Thr27, Glu29 and Leu30. A tetrahedrally coordinated water molecule is a donor to N7 and a phosphate O atom of the nucleotide, and an acceptor of hydrogen bonds from Gly28 (NH) and Thr31 (OH). The side chain of Gln179 interacts with the side chain of Lys20, creating a stabilizing effect on the position of helix α1 and thus on the entire binding site of the inhibitor (Fig. 3 ▸ *a*). Essentially, all of the residues involved in the inter­actions with AMP in muscle FBPase are also observed in this role in the liver isozyme (Gidh-Jain *et al.*, 1994 ▸; Fig. 3 ▸ *b*). However, there are two exceptions. Firstly, in muscle FBPase position 20 is occupied by a positively charged Lys, whereas in the liver enzyme it is occupied by a negative-charge-bearing Glu. The second difference concerns loop L8 (residues 176–179), which in liver FBPase is formed by Ala-Met-Asp-Cys and in muscle FBPase by Ser-Thr-Gly-Gln. The muscle-specific sequence allows the anchoring of the N3 atom of the AMP ligand by a water molecule, which is held in place by the main-chain C=O group of Thr177 and the side chain of Gln179. The water molecule in this network of hydrogen-bond interactions is a bifurcated donor to Thr177 and to another water molecule, and is a linear donor to N3 of AMP.

The most significant difference in the AMP-binding sites between the two isozymes concerns the folding pattern of the N-terminal region (Fig. 2 ▸). In liver FBPase there is a specific N-terminal motif α1–L1–α2 in both the T and the R state. This motif is formed by residues 7–28 and it also exists in muscle FBPase saturated with AMP in its T state. In contrast to the above structures, in the R state of muscle FBPase helix α1 (residues 8–18) is partly converted into β-strand βα1 and the rest of the motif (residues 19–28) is disordered (has poor electron density).

### 3.3. The leucine lock   

**Figure 4:**
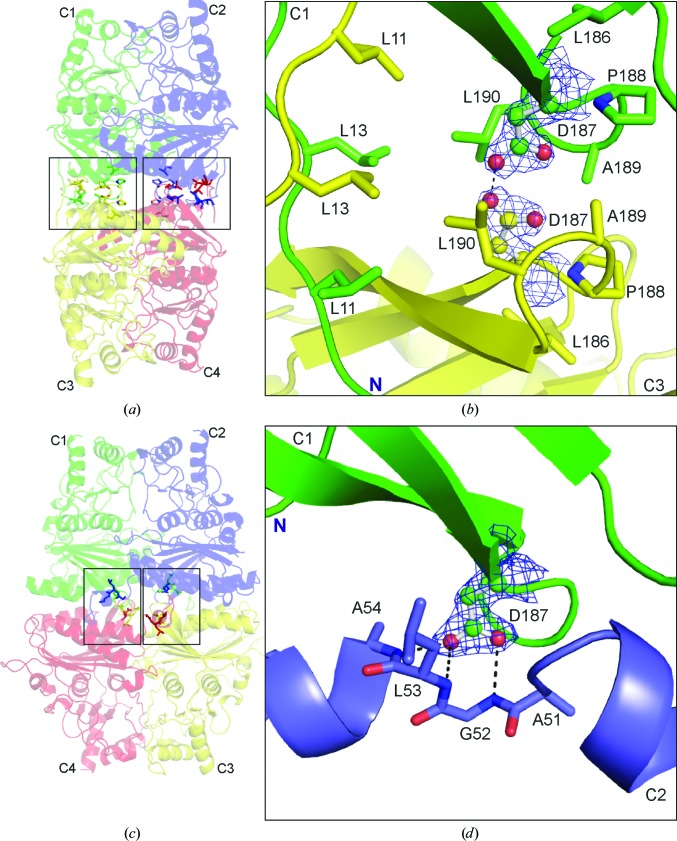
Asp187 and its involvement in subunit interactions, with hydrogen bonds shown as dashed lines. (*a*) A general view of muscle FBPase in the R state, with the two leucine locks between the upper and lower dimers boxed. (*b*) Residues at the interface of subunits C1–C3 in the R state participating in the formation of the leucine lock. The Asp187 residues from subunit C1 (green) and from subunit C3 (yellow) are shown in ball-and-stick representation in 2*F*_o_ − *F*_c_ electron density contoured at the 1.0σ level. (*c*) A general view of muscle FBPase in the T state, with residues at the interface of subunits C1·C2 and C3·C4 boxed. (*d*) Residues at the interface of subunits C1·C2 in the T state participating in the stabilization of loop L2 in the disengaged position. Asp187 (ball-and-stick representation) from subunit C1 (green) is shown in 2*F*_o_ − *F*_c_ electron density contoured at the 1.0σ level. Ala51, Gly52, Leu53 and Ala54 from subunit C2 are shown in blue.

In the R state of muscle FBPase, but not of the liver enzyme, the N-terminal fragments (residues 8–18) of subunits C1–C3 (and also of C2–C4) approach each other and form a hydrophobic region which we term the ‘leucine lock’ (Fig. 4 ▸ *b*). The name reflects the characteristic arrangement of six leucine residues (three from each subunit) across a twofold axis of the tetramer (Figs. 4 ▸ *a* and 4 ▸ *b*). In this motif, Leu11, Leu13 and Leu190 from subunit C1 interact with the same residues from subunit C3, and the same pattern is repeated at the C2–C4 interface. We note that the leucine residues of the lock form a hydrophobic cage shielding the unusual Asp187⋯Asp187 interaction (Fig. 4 ▸ *b*) described in the following section. In the liver isozyme, Leu11 is substituted by asparagine, the polar character of which precludes the formation of a similar hydrophobic motif.

### 3.4. The role of Asp187 in control of the catalytic loop conformation   

In the (catalytically active) R state of muscle FBPase, the upper (C1·C2) and lower (C3·C4) dimers are in a perpendicular (cruciform) orientation stabilized by two leucine locks fixing the interfaces between subunits C1 and C3 and also between C2 and C4 (Table 3 ▸, Fig. 4 ▸ *a*). The hydrophobic leucine lock is not the only interaction motif that stabilizes this conformation. In fact, the lock encages a small volume filled with the side chains of the Asp187 residues from the interacting subunits. The side-chain carboxylates are in an evident O⋯H⋯O hydrogen-bonding contact, with an O^δ2^⋯O^δ2^ distance of 2.40 Å, as confirmed by excellent electron density (Fig. 4 ▸ *b*). Such a short contact, comparable with the strongest O⋯H⋯O hydrogen bonds known (Jaskolski *et al.*, 1978 ▸), is not possible between negatively charged carboxylate anions and unambiguously indicates protonation, or at least hemi-protonation, of the Asp187 side chain. The latter situation is more likely as, in contrast to O^δ2^⋯O^δ2^, the O^δ1^⋯O^δ1^ distance is much longer (3.17 Å). (An interaction of two protonated carboxylic groups would lead to a carboxylic acid dimer with equivalent O⋯O distances.) Although in bulk solvent at pH 7.4 (the pH of crystallization of this form) it is not possible to have a fully protonated carboxylic group (p*K*_a_ of ∼3.9), in the confined environment of the leucine cage two carboxylates could certainly be hemi-protonated to form a hydrogen-bonded anion. The main point of this interaction within the leucine cage of the R form is that it eliminates Asp187 from interactions with any other elements of the protein structure and at the same time provides additional stability for the R-form tetramer.

Upon transition to the (catalytically inactive) T state, however, the leucine lock is disrupted and there is no longer a protective environment for the Asp187⋯Asp187 interaction (Figs. 4 ▸ *c* and 4 ▸ *d*). Exposed to bulk solvent at neutral pH, the aspartates become deprotonated and separate. As the upper subunits rotate +105° relative to the lower subunits, the Asp187 residue of subunit C1 is now found in closer proximity to subunit C4 of the lower dimer. In its characteristic T conformation, the Asp187 carboxylate (of C1) forms a highly specific network of N—H⋯O hydrogen-bond interactions with the amide groups of residues in loop L2 from subunit C2 (Table 4 ▸, Fig. 4 ▸ *d*), fixing the loop in the inactive disengaged conformation. In this state, the L2 loop is unable to participate in the catalytic mechanism.

Thus, the whole sequence of structural transformations within the muscle FBPase tetramer seems to be focused on residue Asp187, which is either able (in the T state) or unable (in the R state) to influence the catalytic loop.

### 3.5. The T-to-R transition   

**Figure 5:**
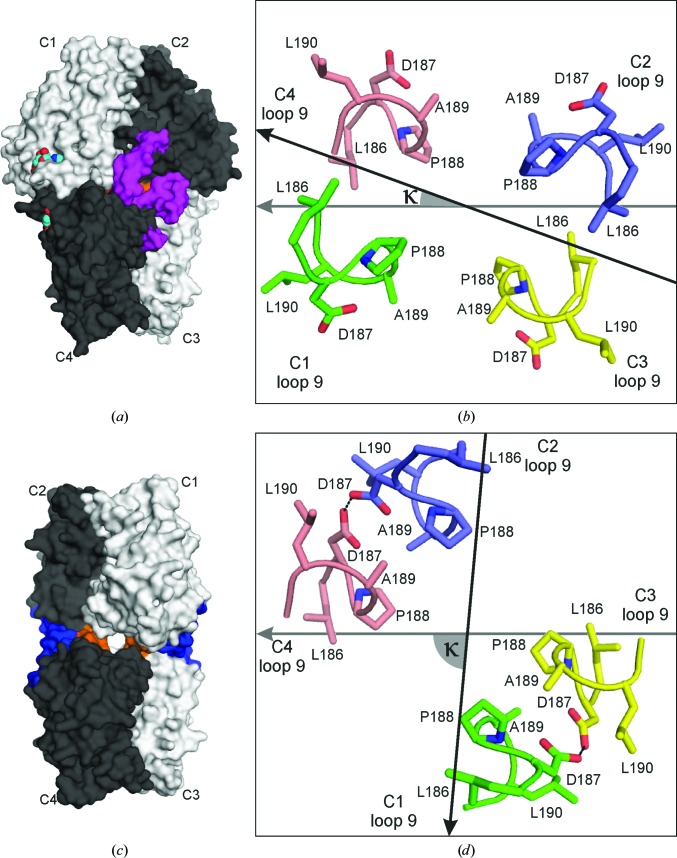
(*a*) and (*c*) show a general view of muscle FBPase in the T state (*a*) and the R state (*c*), with the ‘doorstop’ loops L9 marked in orange. The catalytic loops L2 in (*a*) are marked in magenta and the two leucine locks between the upper and lower dimers in (*c*) are marked in dark blue. The AMP molecules in (*a*) are shown as space-filling models with cyan C atoms. (*b*) and (*d*) show a top view of the residues (186–190) inside the central hydrophobic cavity participating in the formation of the ‘doorstop’ for the T state (*b*) and the R state (*d*). The vectors illustrate the orientation of the upper dimer C2→C1 (black arrow) with respect to the lower dimer C3→C4 (grey arrow), as in Figs. 1 ▸(*c*) and 1 ▸(*d*). The κ angle between these vectors is marked with a grey wedge.

Both the T (κ = +20°) and the R (κ = −85°) conformations are extreme states that are stabilized by steric hindrances specific to each of them. A steric ‘doorstop’ defines the boundaries for the κ rotation between +20 and −85°. This ‘doorstop’ (marked in orange in Figs. 5 ▸ *a* and 5 ▸ *b*) is created by residues from loops L9 (Figs. 5 ▸ *b* and 5 ▸ *d*). Additionally, in the T state, any dimer rotations are blocked by the catalytic loop (marked in violet in Fig. 5 ▸ *a*) in its disengaged position. This hindrance disappears after dissociation of AMP from the allosteric site, when the catalytic loop swings into its engaged position. Even so, the rotation is then possible only in the counterclockwise direction, as clockwise movement is still blocked by loops L9 (Figs. 5 ▸ *a* and 5 ▸ *b*). When the R state is reached, the two leucine locks and the hydrogen bonds between the Asp187 side chains stabilize this form and at the same time hinder further rotation of the dimers (Figs. 5 ▸ *c* and 5 ▸ *d*).

The T-to-R transition is thought to be driven by the release of AMP from the enzyme. We have simulated this transition by subjecting the AMP-containing crystals of T(+AMP) to backsoaking in an Mg^2+^-containing buffer. If too lengthy, the backsoaking process leads to crystal disintegration, strongly indicating that profound structural changes are taking place in the crystal lattice during this process. However, with a quick soak we were able to preserve the crystal integrity and diffraction, although the diffraction quality was significantly degraded. Nevertheless, it was possible to collect X-ray diffraction data and determine the crystal structure, designated T(−AMP) in Table 1 ▸. It showed that the general architecture and crystal packing of the protein molecules was largely unchanged, with notable exceptions, and that the AMP-binding sites were indeed empty. The T(−AMP) structure therefore provides information about the structural changes leading from the canonical T state to the active R state of the muscle enzyme upon the release of AMP.

**Figure 6:**
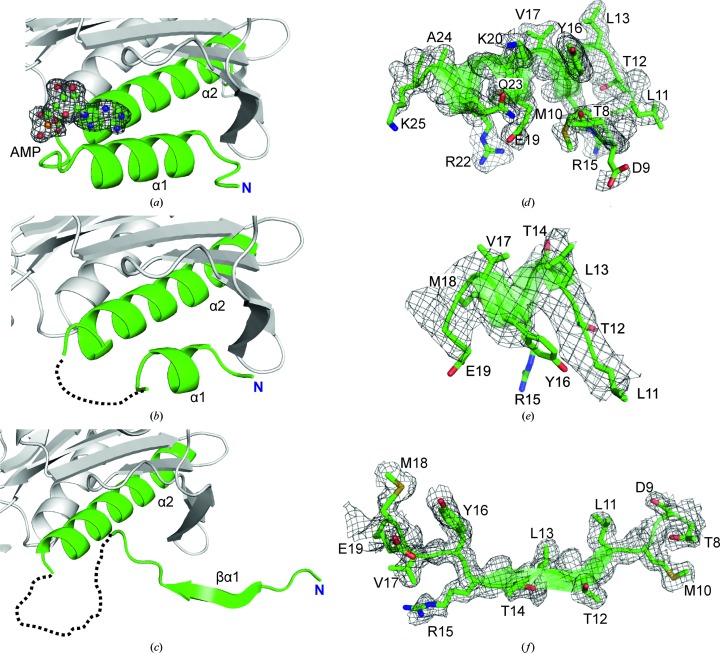
The three stages of refolding of the N-terminal region of muscle FBPase during the T-to-R transition. (*a*) In the T state in complex with AMP (ball-and-stick model) there is a long helix α1 (residues 13–23) at the N-terminus, near the AMP molecule, shown in an *F*_o_ − *F*_c_ OMIT electron-density map (grey mesh) contoured at the 4σ level. (*b*) In the T state, but without AMP, the segment Glu19–Thr31 is characterized by poor electron density (dashed loop omitted from the model) and there is only a short α-helical segment at the N-terminus. (*c*) In the R state the segment Glu19–Thr30 is characterized by poor electron density (dashed loop omitted from the model) and in the middle of the unfolded segment Thr8–Glu19 there is a new β-sheet (βα1) formed by residues Leu11–Arg15. The key N-terminal oligopeptides that change conformation on the T-to-R transition are shown in full atomic representation on the background of transparent secondary-structure elements in annealed *F*_o_ − *F*_c_ OMIT maps in (*d*), (*e*) and (*f*), corresponding to (*a*), (*b*) and (*c*), respectively. The fragments shown on the right and the corresponding map contour levels are Thr8–Lys25 and 4.0σ in (*d*), Leu11–Glu19 and 3.6σ in (*e*) and Thr8–Glu19 and 4.0σ in (*f*), respectively.

The results demonstrated that muscle FBPase still retains a T-like quaternary conformation during the first phase of the T-to-R transition even though the AMP-binding sites are no longer occupied. However, in contrast to the canonical T state (Figs. 6 ▸ *a* and 6 ▸ *d*), residues 19–28 in T(−AMP) show poor electron density (Figs. 6 ▸ *b* and 6 ▸ *e*), which suggests an important role of this region in the organization of the AMP-binding site. Ultimately, as a result of AMP dissociation (*e.g.* from subunit C1), the N-terminal helix α1 is partly transformed in the R state into β-strand βα1 and its interactions with loop L8 are lost (Figs. 6 ▸ *c* and 6 ▸ *f*). Moreover, helices α2 and α3 change their positions, which entails a change of the conformation of the catalytic loop L2 towards its disordered state. In consequence, the hydrogen bonds between the main-chain amides of residues Ala52-Gly53-Leu54 from loop L2 of subunit C1 and Asp187 from C2 are broken, and Asp187 becomes encaged in the leucine lock. As a result, loop L9 (residues 188–191) translocates to the centre of the tetramer, changing the character of the central hydrophobic cavity. The secondary-structure transformations illustrated in electron density in Figs. 6 ▸(*d*), 6 ▸(*e*) and 6 ▸(*f*) are also reflected in the refinement results, where the average *B* factors of the N-terminal fragments are only somewhat higher than for the rest of the structure (Supplementary Table S2).

### 3.6. AMP binding results in improved thermal stability of muscle FBPase   

**Figure 7:**
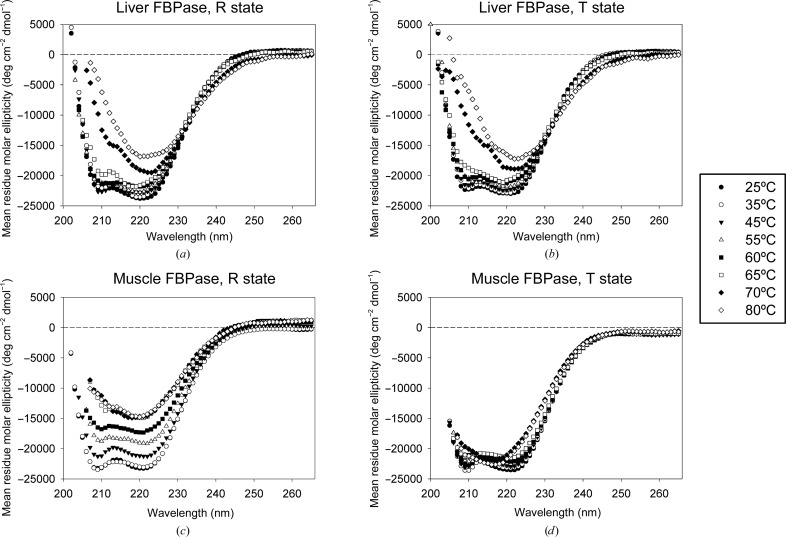
Circular-dichroism spectra of muscle and liver FBPases in R and T states at increasing temperature. The top (*a*, *b*) and bottom (*c*, *d*) panels are for the liver and muscle isozymes, respectively. The left panels (*a*, *c*) correspond to the R state and the right panels (*b*, *d*) to the T state. At 25°C both isozymes in both states exhibit similar spectra. The transition to the T state induced by AMP binding has little effect on the thermal denaturation of liver FBPase, while it dramatically increases the stability of muscle FBPase.

To assess the effect of AMP binding on the stability of muscle FBPase and to compare it with the liver isozyme, we collected a series of CD spectra of both recombinant proteins in their active (R) and inactive (T) states at temperatures increasing from 25 to 80°C (Fig. 7 ▸). At 25°C all studied FBPase forms produce spectra typical for proteins with high α-helical content, with minima around 210 and 220 nm. In the case of liver FBPase, the addition of AMP has little effect on the thermal stability of the protein (Figs. 7 ▸ *a* and 7 ▸ *b*). Both the R and T states of liver FBPase show only very small changes up to 60°C. At temperatures above 65°C the liver isozyme shows signs of destruction of the α-helical structures. Additionally, a general weakening of the signal is observed, which results from aggregation and precipitation of the protein. The behaviour of muscle FBPase is markedly different, both in the R state and, after addition of AMP, in the T state (Figs. 7 ▸ *c* and 7 ▸ *d*). The active R conformation of the muscle enzyme shows evidence of aggregation and precipitation at temperatures as low as 65°C, but signs of destruction of secondary structure are not observed until 70°C. In the T state, a gradual loss of secondary structure is observed at similar temperatures. However, there are no signs of aggregation or precipitation of muscle T-state FBPase even at 80°C, indicating that although the α-helical structures are similarly sensitive to temperature in both the R and T states, the quaternary structure of muscle FBPase in the T form does not undergo destruction at elevated temperatures.

To corroborate the above findings, the melting temperatures for muscle FBPase in the R and T states were determined by turbidimetry. In agreement with the CD data, the turbidimetry measurements show that the active R conformation of muscle FBPase is highly sensitive to increasing temperature. We found that the R state has a melting temperature of 70°C (Supplementary Fig. S1). In the case of the T state, we were not able to determine the melting point because there was no significant precipitation even at 80°C (Supplementary Fig. S1), indicating that the melting temperature is higher.

The heat-stability profiles of the various forms of FBPase seem to reflect the differences in the quaternary structure. The R state of muscle FBPase, which shows the highest sensitivity to thermal denaturation, has the least compact structure and the largest solvent-accessible surface. The T-to-R transition is coupled with only a minimal change of the total dimer–dimer interaction area (Table 5 ▸). This is the case because although the transition drastically reduces the area of the C1–C4 (and C2–C3) surface, the formation of the leucine locks stabilizes the tetramer as a whole by the formation of new interaction interfaces between subunits C1 and C3 (and C2–C4). In such an R-state conformation, relatively little thermal energy may be required to expose the hydrophobic core residues to water, which explains why muscle FBPase in the R state undergoes aggregation at relatively low temperatures. Binding of AMP and transition to the much more compact T state prevents the aggregation of muscle FBPase and increases its thermal stability. It should be noted that the numbers in Table 5 ▸ have to be taken with a generous margin of error because in the different conformational forms different stretches of oligopeptide sequences are absent from the coordinate files.

The markedly different behaviour of thermal stability on R-to-T transition of the two isozymes can be correlated with the dramatic difference in the corresponding structural (quaternary) transitions. The R-to-T transition of liver FBPase requires only a minimal κ rotation of ∼13° (clockwise from 0 to +13°), explaining the moderate change of thermal stability upon this transformation. In contrast, AMP binding to muscle FBPase induces a κ rotation of +105° (clockwise from −85° to +20°), explaining the sharp increase in thermal stability.

## 4. Discussion   

The most important finding of this study is the observation that muscle FBPase adopts a unique, cross-like quaternary arrangement of its subunits in the active R state, in which the upper dimer is rotated by nearly −90° with respect to the lower dimer. This unexpected quaternary conformation has also been confirmed by SAXS experiments conducted under more physiological conditions (using 50 m*M* HEPES buffer and in the absence of salt), which demonstrated that in the absence of AMP the enzyme exists in the cruciform arrangement of the subunits, whereas the addition of AMP induced the formation of a T-like state (Szpotkowski, personal communication). Thus, whereas the quaternary structure of the inactive T form of muscle FBPase closely resembles the corresponding conformation of the liver isozyme, the cruciform R state of muscle FBPase is dramatically different from the planar arrangement observed for the active liver homotetramer.

The cruciform R state is also entirely different from the essentially planar structure presented as the ‘R state’ by Shi *et al.* (2013 ▸). A comparison of the N-terminal secondary-structure elements of the latter model with the structures presented here (Supplementary Table S1) suggests, however, that Shi and coworkers observed not the properly formed R-state FBPase but the first step in the T-to-R transition, which we define as the FBPase structure in the T state without AMP, T(−AMP). Presumably, the form described as the R state by Shi and coworkers was crystallized in the presence of traces of AMP used during the protein-purification procedure. Such a suspicion is supported by the kinetic data presented by Shi and coworkers, indicating a very low cooperativity of the inhibition of FBPase by AMP. The reported Hill coefficient was equal to 1.3 instead of 2, which strongly suggests that the FBPase sample used in the reported experiments was partially complexed with this inhibitor.

One of the consequences of such a cruciform structure of the muscle FBPase R form is the exposure of new solvent-accessible surfaces, which in the T state were involved in the formation of intersubunit interfaces between the lower and upper dimers. The availability of new structural elements, for example of new surfaces, for protein–protein interactions may explain the fundamentally different abilities of the two FBPase isozymes to interact with various binding partners (Dzugaj, 2006 ▸).

In contrast to the liver enzyme, muscle FBPase plays a complex role in cell physiology, affecting, through noncatalytic activities, the progression of the cell cycle and ensuring ATP production by mitochondria under stress conditions (Gizak *et al.*, 2012 ▸). It has been shown that these functions of muscle FBPase depend on its association with a set of cellular proteins and that the active R-like state of the isozyme is required for the interactions; only muscle FBPase in the R conformation is capable of association with mitochondria and can protect them against accelerated glycolysis-related stress signals (Pirog *et al.*, 2014 ▸).

The extreme rotation of the dimers in the R state of the muscle isozyme correlates with the complete loss of all of the interactions between the C1–C4 (and C2–C3) subunits that are observed in the T state. In the muscle-specific R state, the only interactions between the upper and the lower dimers are formed between subunits C1 and C3 (and also between C2 and C4). This suggests that the AMP-saturated T state of muscle FBPase is energetically favourable and, as a consequence, that the stabilization of the inactive T form is much stronger than that of the active R form. This structural conclusion has been confirmed by the results of heat-denaturation experiments and melting-temperature determination, which showed that AMP binding to muscle FBPase significantly increases the stability of the enzyme. In contrast to muscle FBPase, the liver enzyme shows similar thermal stability in the R and T states.

Nonetheless, in the absence of AMP, muscle FBPase is fully active and there are no basic differences in the catalytic properties between the muscle and liver isozymes. This raises an important question about the mechanism that stabilizes the active conformation of muscle FBPase. Based on the structural data, it might be hypothesized that a crucial role in the stabilization of the R state is played by the residues forming the leucine lock between subunits C1 and C3 (and between C2 and C4). This unique hydrophobic nucleus consisting of six leucine side chains (Leu11, Leu13 and Leu190 from each subunit) is disrupted in the T state, which emphasizes the role of hydrophobic interactions across the tetramer in the stabilization of the R form of the enzyme. Even more importantly, the leucine lock motif between subunits C1 and C3 encages a tandem of Asp187 residues from the interacting subunits, which are paired by a strong hydrogen bond, after at least partial (hemi) protonation, to additionally stabilize the R form. When, upon the R-to-T transition, the leucine lock is broken, the Asp187 residues are no longer isolated, become fully deprotonated and change their conformation to form hydrogen bonds stabilizing the catalytic loop L2 in its dis­engaged (inactive) conformation. Specifically, the Asp187 carboxylate group of subunit C1 forms three N—H⋯O hydrogen bonds to three consecutive main-chain amide groups in the L2 sequence of subunit C2.

The relatively weaker stabilization of the R form of muscle FBPase seems to be reflected in the secondary-structure content of the enzyme. This form, in contrast to the T state of muscle FBPase and to all known forms of the liver isozyme, contains a significant proportion of elements that are unstructured and/or have poorly defined electron density (Table 2 ▸). Intriguingly, most of those elements are found in the region of the AMP-binding site. At present, the mechanism by which AMP induces the formation of its binding site, which involves the folding of helix α1 and the proper spatial organization of loops L1, L5 and L6, is elusive. However, to the best of our knowledge, the formation of this binding site in muscle FBPase in an α→β transition is the first example of a reversible refolding of structural elements observed in a conserved, rigid protein involved in basic energy metabolism.

The presence in muscle FBPase of a region that has different secondary structure in two different enzyme states raises the question about the sequence of structural changes leading to allosteric inhibition by AMP. In one scenario, muscle FBPase might function according to the concerted model of allosteric inhibition, in which the R and T forms are in equilibrium in the absence of any effectors and the binding of AMP shifts the equilibrium towards the T conformation. In another scenario, binding of AMP would be the trigger for conformational changes leading to the T form. In the former scenario, the organization of the AMP-binding site would be the result of the global R-to-T rotation. In the latter model, AMP would be the trigger inducing the transition of the entire protein architecture into the T state. To test the plausibility of the two models, we briefly soaked (10 s) well formed crystals of AMP-saturated, *i.e.* T-state [T(+AMP)], FBPase with magnesium (100 m*M*), which is known to compete indirectly with AMP for the binding to the enzyme. As a result, we observed the enzyme in the T form but with the AMP site empty [T(−AMP)] and partially unstructured. Therefore, in conclusion, it is evident that the transition from the inactive to the active state of the tetramer starts with a disruption of the allosteric site. By analogy, the opposite process of the formation of the AMP-binding site within the R structure is the first step during the R-to-T transition. In other words, the transition between these two states is a discrete, triggered conformational jump, rather than a smooth shift of an equilibrium.

An alternative model of the T-to-R transition assuming subunit exchange *via* dissociation/re-association of the upper and lower dimers has been proposed for mammalian FBPase by Nelson *et al.* (2001 ▸). However, the rate of such a subunit exchange is relatively slow [both for the liver (Nelson *et al.*, 2001 ▸) and muscle FBPase (unpublished work)] and there is no dilution effect on the activity of FBPase. Taken together, these observations make the ‘dimer-exchange’ scenario highly improbable.

## 5. Conclusions and outlook   

In the present paper, we have demonstrated that the R state of mammalian muscle FBPase is significantly different from the corresponding form of the liver isozyme and that this novel quaternary structure may explain the complex role of muscle FBPase in cell physiology. The presented structural description of the new protein surfaces exposed in the R state may serve as a starting point for the identification of the cellular binding partners of muscle FBPase and for the design of small-molecule competitors for the interactions.

We have also presented evidence that muscle FBPase belongs to the family of proteins containing unstructured, intrinsically disordered elements that adopt a highly ordered structure upon interaction with physiological factors (here after interaction with AMP). Some level of structural flexibility and local fluctuations in a native state has previously been reported for adenylate kinase (Rundqvist *et al.*, 2009 ▸; Schrank *et al.*, 2009 ▸). However, to the best of our knowledge, muscle FBPase is the first example among the basic meta­bolism enzymes that has been shown to contain structural elements that undergo conformational rearrangements affecting the structure at the secondary-to-quaternary level under native conditions.

While the present series of muscle FBPase structures has revealed fascinating aspects of the quaternary and secondary structure of the enzyme in relation to its function, there are still a number of puzzling questions about this unusual protein. One concerns the mechanism by which its association with aldolase abrogates the inhibitory effect of AMP, *i.e.* whether the binding of aldolase to FBPase fixes the R state or occludes the AMP-binding site.

The cruciform structure of the R state presented in this work shows the exposure of unexpected protein surfaces for protein–protein interactions, which may be the starting point for future analyses. Crystallization experiments are in progress to obtain an FBPase–aldolase complex. Another question concerns the details of the catalytic mechanism and the synergistic inhibition by allosteric (AMP and NAD^+^) and competitive (fructose 2,6-bisphosphate) effectors. Work, including X-ray crystallography and SAXS, is also in progress on structural and biochemical characterization of specific, site-directed FBPase mutants.

## Supplementary Material

PDB reference: human muscle fructose-1,6-bisphosphatase in R state, 5et5

PDB reference: in T state, AMP complex, 5et6

PDB reference: in T state without AMP, 5et7

Supporting information.. DOI: 10.1107/S2059798316001765/dw5156sup1.pdf

Raw X-ray diffraction images corresponding to the T(+AMP) data set. URL: http://dx.doi.org/10.18150/8324764

Raw X-ray diffraction images corresponding to the T(−AMP) data set. URL: http://dx.doi.org/10.18150/10.18150/2374334

Raw X-ray diffraction images corresponding to the R data set. URL: http://dx.doi.org/10.18150/6428373

## Figures and Tables

**Table 1:** Data-collection and refinement statistics Values in parentheses are for the highest resolution shell.

FBPase state	R	T(−AMP)	T(+AMP)
Data collection			
Radiation source	BESSY II, Berlin	BESSY II, Berlin	BESSY II, Berlin
Beamline	14.1	14.2	14.2
Wavelength (Å)	0.91841	0.91841	0.82657
Temperature (K)	100	100	100
Space group	*I*4_1_22	*C*222	*C*222
Unit-cell parameters (Å)	*a* = *b* = 72.57, *c* = 235.35	*a* = 218.48, *b* = 234.77, *c* = 71.85	*a* = 218.61, *b* = 234.73, *c* = 71.61
Resolution (Å)	36.28–1.67 (1.77–1.67)	45.46–2.99 (3.17–2.99)	45.39–1.84 (1.96–1.84)
Reflections (collected/unique)	482798/37062	461483/37769	789342/156646
Completeness (%)	99.8 (99.4)	99.4 (97.4)	99.2 (97.4)
Multiplicity	13.03 (13.18)	12.22 (12.01)	5.04 (4.98)
*R*_merge_ †	0.052 (0.898)	0.120 (0.844)	0.055 (0.693)
〈*I*/σ(*I*)〉	29.89 (3.08)	24.2 (4.03)	19.43 (2.99)
Refinement			
Unique reflections (work)	36061	36769	155565
Test reflections	1001	1000	1081
Matthews volume (Å^3^ Da^−1^)	2.07	3.62	3.06
Solvent content (%)	40.5	66.0	59.9
No. of non-H atoms			
Protein	2180	8589	9709
Ligand	—	—	92
Solvent	133	—	664
*R*_work_/*R*_free_ (%)	19.33/21.96	17.70/24.43	16.84/19.21
R.m.s.d. from ideal geometry			
Bond lengths (Å)	0.019	0.015	0.020
Bond angles (°)	1.791	1.767	1.721
Ramachandran statistics (%)			
Favoured	97.8	92.3	97.9
Allowed	2.2	6.6	2.1
PDB code	5et5	5et7	5et6

† *R*_merge_ = 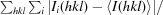 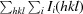, where *I_i_*(*hkl*) is the intensity of observation *i* of reflection *hkl*.

**Table 2:** *DSSP* (Kabsch & Sander, 1983 ▸) assignment of secondary-structure elements in human muscle FBPase in the three states described in this work Gaps in the sequence (…) indicate fragments that were not modelled.

	Residue range		
Secondary-structure element	R state	T state (−AMP)	T state (+AMP)
Allosteric domain			
N-terminal	8–10	11–12	8–12
α1	—	13–19	13–23
βα1	11–15	—	—
L1	16–19…28	20…29	24–28
α2	29–49	30–49	29–48
L2 (catalytic loop)	50–51…73	50…72	49–63…70
αL2	—	—	53–56
α3	74–87	73–86	71–86
L3	88–90	87–90	
β1	91–96		
L4	97–109	97–112	97–109
βL4	103–104	99–104	103–104
αL4 (3_10_-helix)	107–109		
β2	110–121	113–118	110–121
L5	122…129–131	119–122…130–131	122–131
αL5 (3_10_-helix)	—	—	123–128
β3	132–140	132–139	132–140
L6	141…147–160	140–158	141–142…147–160
αL6a (3_10_-helix)	150–152	149–151	149–152
αL6b (3_10_-helix)	156–158		
β4	161–167	159–167	161–167
L7	168–170		
β5	171–176		
L8	177–181		
β6	182–187		
L9	188–191		
β7	192–197		
Catalytic domain			
L10	198–207		
β8	208–210	208–209	208–210
L11	211–220	210–220	211–220
αL11 (3_10_-helix)	213–218	213–215	213–218
α4	221–231		
L12	232–240		
β9	241–242		
L13	243–247		
α5	248–258		
L14	259–260		
β10	261–264		
L15	265–280		
α6	281–290		
L16	291–293		
β11	294–296		
L17	297–315		
αL17 (3_10_-helix)	302–304		
β12	316–319		
T1	320		
α7	321–332	321–335	
C-terminal	333–334	336–337	336–337

**Table 3:** Hydrogen bonds between residues of subunits C1 and C3 in the R state

C1	Distance (Å)	C3
Thr8	2.71	Tyr16
Asp9	3.35	Asn35
Met10	3.54	Glu192
Leu11	2.87	Phe193
Thr12	2.82	Thr14
Thr12	3.75	Thr14
Thr14	3.06	Thr12
Tyr16	2.96	Met10
Thr39	3.71	Asp9
Lys42	3.98	Asp9
Asp187	2.40	Asp187
Asp187	3.17	Asp187
Phe193	2.83	Asp9
Leu195	2.85	Leu11

**Table 4:** Interactions between residues of subunits C1 and C2 in the T state and the R state

T state (+AMP)			R state		
C1	Distance (Å)	C2	C1	Distance (Å)	C2
Arg49	3.14	Gly168			
Arg49	2.76	Ser169	Arg49	3.12	Ser169
Gly52	2.97	Asp187			
Leu53	3.22	Asp187			
Ala54	2.89	Asp187			
Cys128	3.19	Tyr258			
Ser131	3.90	Leu129			
Ser169	3.09	Leu129			
Gly214	3.86	Tyr209			
Lys231	3.10	Glu213	Lys231	3.61	Glu213
Ala242	3.01	Asn212	Ala242	2.93	Asn212
Arg243	3.46	Ser124			
Tyr244	2.75	Tyr244	Tyr244	2.89	Tyr244
Tyr258	2.58	Ser124			

**Table 5:** Subunit and oligomer surface areas and surface areas buried upon oligomerization The surface areas and changes in solvent free energy were estimated in *PDBePISA* (Krissinel & Henrick, 2007 ▸).

Subunit	Oligomer	Solvent-accessible surface (Å^2^)	Surface buried upon interaction (Å^2^)	Change in solvent free energy (kcal mol^−1^)
R				
C1	—	14693	—	—
	C1·C2†	26111	1638	−25.5
	C1·C3‡	26559	1413	−17.5
	C1·C4§	29128	127	1.2
	(C1·C2†)–(C3‡·C4§)	46063	3079	−32.5
T(+AMP)				
Tetramer I				
C1	—	14814	—	—
	C1·C2¶	24998	2306	−32.4
C4	—	14895	—	—
	C3††·C4	25225	2270	−31.6
	C1–C3††	28848	405	−8.5
	C1–C4	27314	1183	−6.0
	C3††–C2¶	27314	1183	−6.0
	C2¶–C4	28848	405	−8.5
	(C1·C2¶)–(C3††·C4)	44063	3081	−24.6
Tetramer II				
C1	—	14718	—	—
	C1·C2‡‡	24783	2317	−30.2
C4	—	14835	—	—
	C3§§·C4	25047	2299	−32.8
	C1–C3§§	28692	408	−8.2
	C1–C4	27182	1170	−4.5
	C3§§–C2‡‡	27182	1170	−4.5
	C2‡‡-C4	28692	408	−8.2
	(C1·C2‡‡)–(C3§§·C4)	43701	3065	−21.2

† Subunit C2 is a symmetrical copy of C1 generated by (*y*, *x*, −*z*), twofold axis along [110].

‡ Subunit C3 is a symmetrical copy of C1 generated by (−*y*, −*x*, −*z*), twofold axis along [1−10].

§ Subunit C4 is a symmetrical copy of C1 generated by (−*x*, −*y*, *z*), twofold axis along [001].

¶ Subunit C2 is a symmetrical copy of C1 generated by (−*x*, *y*, −*z*), twofold axis along [010].

†† Subunit C3 is a symmetrical copy of C4 generated by (−*x*, *y*, −*z*), twofold axis along [010].

‡‡ Subunit C2 is a symmetrical copy of C1 generated by (*x*, −*y*, 1 − *z*), twofold axis along [100].

§§ Subunit C3 is a symmetrical copy of C4 generated by (*x*, −*y*, 1 − *z*), twofold axis along [100].
