# Supplementary material for: T-to-R switch of muscle fructose-1,6-bisphosphatase involves fundamental changes of secondary and quaternary structure
Source: Acta Crystallogr D Struct Biol. 2016 Mar 30;72(Pt 4):536–50. doi: 10.1107/S2059798316001765 (PMC4822563; doi:10.1107/S2059798316001765)
Supplement: Supplementary file 1 [file d-72-00536-sup1.pdf]

STRUCTURAL BIOLOGY

Volume 72 (2016)

Supporting information for article:

T-to-R switch of muscle FBPase involves fundamental changes of secondary and quaternary structure

Jakub Barciszewski, Janusz Wisniewski, Robert Kolodziejczyk, Mariusz Jaskolski, Dariusz Rakus and Andrzej Dzugaj

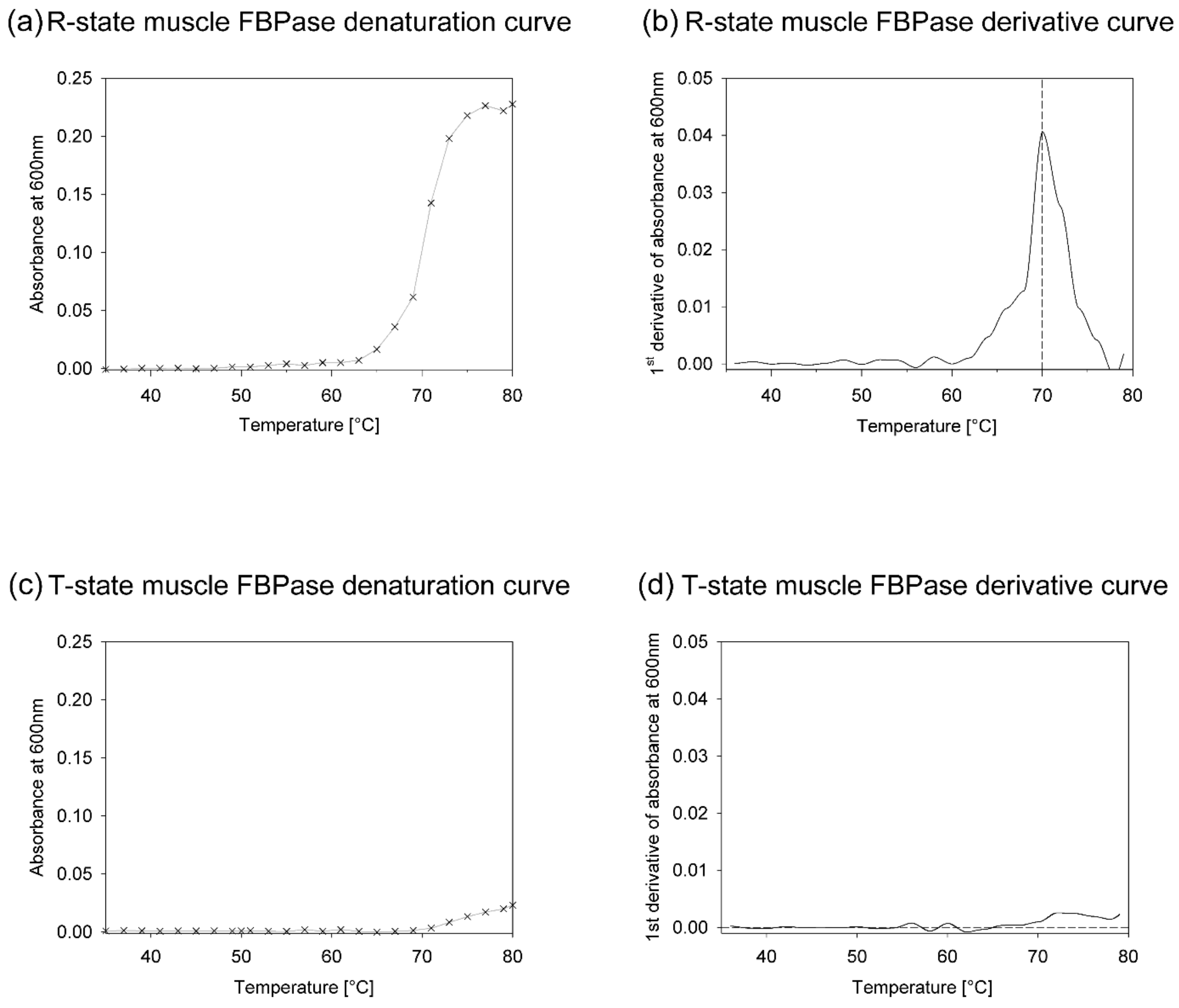

Figure S1 Turbidimetric thermal denaturation and derivative curves for muscle FBPase. The denaturation curves were obtained by linear interpolation of the experimental data. The derivative curves were calculated using Savitzky-Golay filter (Savitzky & Golay, 1964). The protein in the R-state begins to precipitate at 65°C (a) with a melting temperature of 70°C (b). In the T-state, the protein does not begin to precipitate until 70°C and shows only minimal precipitation between 70°C and 80°C (c and d).

Table S1 DSSP (Kabsch & Sander, 1983) assignment of secondary structure elements in the crystal structures of human muscle FBPase in T-state without AMP [T(–AMP)] described in this work and in wild type human muscle FBPase (PDB ID 4HE0) described previously by Shi *et al.* (2013).

Gaps in the sequence (…) indicate fragments that were not modeled in electron density.

**Secondary structure element**	**Residue range**	
	**4HE0**	**T(-AMP)**
**Allosteric domain**		
N-terminal	7-12	11-12
α1	13-20	13-19
βα1	-	-
L1	21-23…27-28	20…29
α2	29-49	30-49
L2 (*catalytic loop*)	50…71	50…72
αL2	-	-
β1L2	59-63	-
β2L2	69-71	-
α3	72-86	73-86
L3	87-90	87-90
β1	91-96	91-96
L4	97-112	97-112
βL4	99-104	99-104
αL4 (3_{10} *helix*)	107-109	107-109
β2	113-121	113-118
L5	122-131	119-122…130-131
αL5 (3_{10} *helix*)	123-125	-
β3	132-140	132-139
L6	141-160	140-158
αL6a (3_{10} *helix*)	149-152	149-151
αL6b (3_{10} *helix*)	156-158	156-158
β4	161-167	159-167
L7	168-170	168-170
β5	171-176	171-176
L8	177-180	177-181
β6	181-187	182-187
L9	188-191	188-191
β7	192-200	192-197

**Catalytic domain**		
L10	201-207	198-207
β8	208-210	208-209
L11	211-220	210-220
αL11 (3_{10} *helix*)	213-218	213-215
α4	221-231	221-231
L12	232-240	232-240
β9	241-242	241-242
L13	243-247	243-247
α5	248-258	248-258
L14	259-260	259-260
β10	261-264	261-264
L15	265-280	265-280
α6	281-290	281-290
L16	291-293	291-293
β11	294-296	294-296
L17	297-315	297-315
αL17 (3_{10} *helix*)	302-304	302-304
β12	316-319	316-319
T1	320	320
α7	321-332	321-335
C-terminal	333-335	336-337

Table S2 Comparison of average B-factors (Å^2) of the N-terminal fragment vs. total structure of the three states of FBPase.

**<B>**	**R**	**T(–AMP)**	**T(+AMP)**
N-terminal/Total	43 / 36	110 / 74	44 / 41

References

Kabsch, W. & Sander, C. (1983). *Biopolymers* **22**, 2577-2637.

Savitzky, A. & Golay, M.J.E. (1964). *Anal. Chem.* **36**, 1627-1639.

Shi, R., Chen, Z-Y., Zhu, D-W., Li, C., Shan, Y., Xu, G. & Lin, S-X. (2013). *PLoS One* **8**, e71242.
